# Comparison of CRISPR/Cas Endonucleases for *in vivo* Retinal Gene Editing

**DOI:** 10.3389/fncel.2020.570917

**Published:** 2020-09-10

**Authors:** Fan Li, Kristof Wing, Jiang-Hui Wang, Chi D. Luu, James A. Bender, Jinying Chen, Qi Wang, Qinyi Lu, Minh Thuan Nguyen Tran, Kaylene M. Young, Raymond C. B. Wong, Alice Pébay, Anthony L. Cook, Sandy S. C. Hung, Guei-Sheung Liu, Alex W. Hewitt

**Affiliations:** ^1^Menzies Institute for Medical Research, University of Tasmania, Hobart, TAS, Australia; ^2^State Key Laboratory of Ophthalmology, Zhongshan Ophthalmic Centre, Sun Yat-sen University, Guangzhou, China; ^3^Centre for Eye Research Australia, Royal Victorian Eye and Ear Hospital, East Melbourne, VIC, Australia; ^4^Ophthalmology, Department of Surgery, The University of Melbourne, Parkville, VIC, Australia; ^5^Wicking Dementia Research and Education Centre, University of Tasmania, Hobart, TAS, Australia; ^6^Department of Ophthalmology, The First Affiliated Hospital of Jinan University, Guangzhou, China; ^7^Department of Surgery, Royal Melbourne Hospital, The University of Melbourne, Parkville, VIC, Australia; ^8^Department of Anatomy and Neuroscience, The University of Melbourne, Parkville, VIC, Australia

**Keywords:** CRISPR (clustered regularly interspaced short palindromic repeats), retina, retinal dystrophy, gene editing, AAV (adeno-associated virus)

## Abstract

CRISPR/Cas has opened the prospect of direct gene correction therapy for some inherited retinal diseases. Previous work has demonstrated the utility of adeno-associated virus (AAV) mediated delivery to retinal cells *in vivo*; however, with the expanding repertoire of CRISPR/Cas endonucleases, it is not clear which of these are most efficacious for retinal editing *in vivo*. We sought to compare CRISPR/Cas endonuclease activity using both single and dual AAV delivery strategies for gene editing in retinal cells. Plasmids of a dual vector system with SpCas9, SaCas9, Cas12a, CjCas9 and a sgRNA targeting *YFP*, as well as a single vector system with SaCas9/YFP sgRNA were generated and validated in YFP-expressing HEK293A cell by flow cytometry and the T7E1 assay. Paired CRISPR/Cas endonuclease and its best performing sgRNA was then packaged into an AAV2 capsid derivative, AAV7m8, and injected intravitreally into CMV-Cre:Rosa26-YFP mice. SpCas9 and Cas12a achieved better knockout efficiency than SaCas9 and CjCas9. Moreover, no significant difference in *YFP* gene editing was found between single and dual CRISPR/SaCas9 vector systems. With a marked reduction of YFP-positive retinal cells, AAV7m8 delivered SpCas9 was found to have the highest knockout efficacy among all investigated endonucleases. We demonstrate that the AAV7m8-mediated delivery of CRISPR/SpCas9 construct achieves the most efficient gene modification in neurosensory retinal cells *in vivo*.

## Introduction

Being discovered as a critical component of some bacterial and archaea, acting to counter viral intrusion (Jinek et al., 2012), the Clustered Regularly Interspaced Short Palindromic Repeats (CRISPR)/CRISPR-associated protein (Cas) system has been successfully repurposed for efficient genome editing in mammalian cells (Cong et al., 2013; Mali et al., 2013). This has opened the door to direct gene correction therapy for many inherited retinal diseases. Nevertheless, one of the greatest challenges is the efficient delivery of the CRISPR/Cas genome-editing system to the target tissues or cells in living organisms. Due to the large size of the commonly used SpCas9 (*Streptococcus pyogenes*, ∼4.2 kb) and the loading capacity of some currently available viral vectors for ocular gene therapy such as adeno-associated virus (AAV), recent studies have demonstrated that a dual AAV2 system can be used to deliver CRISPR/Cas9 to effectively perform DNA editing in retinal cells in adult mice (Bakondi et al., 2016; Hung et al., 2016; Latella et al., 2016; Ruan et al., 2017; Yu et al., 2017; Li et al., 2019). Despite the success of this dual-vector strategy, it is challenging to transduce two AAVs into one cell and clearly activity of the CRISPR/Cas machinery requires the receipt of both the endonuclease and sgRNA expression cassettes.

With the expanding repertoire of CRISPR/Cas endonucleases, various CRISPR/Cas systems have been developed that utilize smaller Cas endonuclease from different bacterial species, such as Cas12a (*Acidaminococcus*,∼3.9 kb or *Lachnospiraceae*, ∼3.7 kb), SaCas9 (*Staphylococcus aureus*, 3.2 kb), CjCas9 (*Campylobacter jejuni*, 2.9 kb), NmCas9 (*Neisseria meningitidis*, ∼3.2 kb), making it possible to use a single vector to package both the Cas endonuclease and its sgRNA. A handful of studies have reported the successful *in vivo* genome editing of SaCas9 (Maeder et al., 2019), CjCas9 (Kim et al., 2017; Jo et al., 2019), Cas12a (Koo et al., 2018), and NmeCas9 (Xia et al., 2018) in retinal cells. These various CRISPR/Cas systems differ in their editing efficacy, packageability and protospacer-adjacent motif (PAM) requirement (listed in Supplementary Table S2), which largely expands the *in vivo* application of CRISPR/Cas based genome editing in various tissues or cells. There have been a small number of studies, which have applied all-in-one AAV vector-mediated CRISPR/Cas genome editing in different cells including retinal pigment epithelium cells. Eunji and colleagues reported the successful disruption of the *Vegfa* or *Hif1a* genes in mouse RPE cells using single AAV-CjCas9 (Kim et al., 2017). Other groups have utilized a single AAV vector to deliver SaCas9, or NmeCas9 to a variety of somatic tissue in mice (Ran et al., 2015; Ibraheim et al., 2018; Jarrett et al., 2018; Pan et al., 2018; Xu et al., 2019). Despite the encouraging *in vivo* application of these CRISPR/Cas systems, delivered via dual or all-in-one vectors, it is not clear which are the most efficacious for retinal editing *in vivo*.

The aim of this study was to directly compare the CRISPR/Cas endonuclease activity of single/dual AAV strategies for retinal gene editing in the transgenic mice expressing a yellow fluorescent protein (YFP) reporter. To achieve this, we designed YFP-targeting sgRNAs for each Cas endonuclease and quantified the editing efficiency, indicated by the disruption of YFP *in vitro* and *in vivo*.

## Materials and Methods

### Ethics Approval and Animal Maintenance

All experimental studies were performed in accordance with the Association for Research in Vision and Ophthalmology Statement for the Use of Animals in Ophthalmic and Vision Research and the requirements of the National Health and Medical Research Council of Australia (Australian Code of Practice for the Care and Use of Animals for Scientific Purposes). This study was approved by the Animal Ethics Committees of the University of Tasmania (Reference Number A0014827). CMV-Cre and Rosa26-YFP transgenic mouse lines were maintained on a C57BL/6 background and intercrossed to generate experimental offspring that were heterozygous for each transgene. Adult (8–12 weeks old) CMV-Cre:Rosa26-YFP transgenic mice (YFP mice), which express YFP throughout the retina, were maintained and bred at the University of Tasmania (Hobart, TAS, Australia). Animals were group housed with same-sex littermates in Optimice micro-isolator cages (Animal Care Systems, Centennial, CO, United States) with uninhibited access to food and water. They were maintained on a 12 h light (50 lux illumination) and 12 h dark (<10 lux illumination) cycle, at 20°C to minimize possible light-induced damage to the eye.

### Design and Construction of Cas Endonucleases and sgRNAs Vectors

**FIGURE 1:**
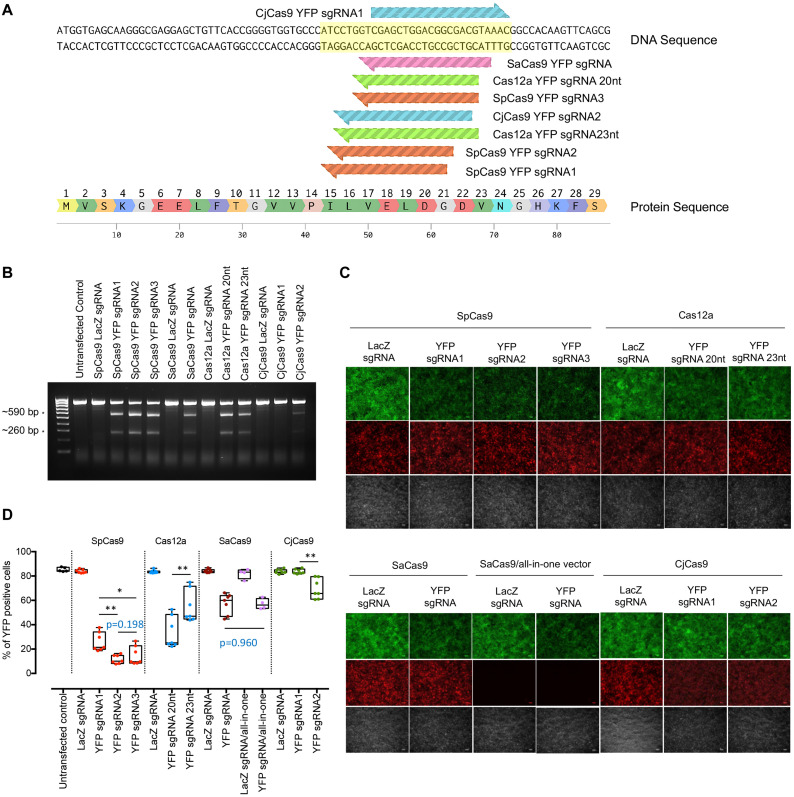
*In vitro* YFP sgRNA validation and selection. **(A)** *YFP*-targeting sequence for sgRNA design. *YFP*-targeting sgRNAs were designed (3 sgRNAs for SpCas9, 1 sgRNA for SaCas9, 2 sgRNAs for Cas12a, and 2 for CjCas9). **(B)** T7E1 assay to detect cleavage efficiency for *YFP*. Expected cleavage products by T7E1 were detected in 2% TAE gel. * Cleavage products around 590 and 260 bp. **(C)** Representative fluorescence microscopy images showing YFP expression in cells transfected with different CRISPR/Cas constructs. Scale bar: 100 μm. **(D)** Flow cytometry analysis for sgRNA selection. Data are represented as mean ± SEM for 4–7 independent replicates. Intergroup comparisons were performed using a one-way ANOVA and corrected for multiple comparisons. HEK293A cells without YFP expression were also included as negative control. No significant difference in *YFP* editing was observed between single and dual CRISPR/SaCas9 vector systems (*p* = 0.9608). Selected sgRNAs for *in vivo* testing were SpCas9 YFPsgRNA2, Cas12a YFP sgRNA20nt, and CjCas9 YFPsgRNA2. *p < 0.05, **p < 0.01.

Single guide RNAs targeting the same 5′ region of the *YFP* gene were designed using a CRISPR design tool^1^ with different relevant PAM sites (Figure 1A). Briefly, three sgRNAs for SpCas9 (referred as SpCas9-YFP sgRNA1, 2, and 3), two sgRNAs with different lengths for Cas12a (referred as Cas12a-YFP sgRNA 20 and 23 nt), two sgRNAs for CjCas9 (referred CjCas9-YFP sgRNA1 and 2) and one sgRNA for SaCas9 (referred as SaCas9-YFP sgRNA, as only one possible PAM site was found in that region) were designed. These sgRNAs were then cloned into the AAV-U6-sgRNA-hSyn-mCherry vector (Addgene #87916). A control sgRNA, targeting the *LacZ* gene (5′-TGCGAATACGCCCACGCGAT-3′), was designed based on a previous study by Swiech et al. (2015) and LacZ sgRNA plasmids were generated and used for *in vitro* validation.

Cas endonuclease plasmids were generated following different cloning approaches. The AAV-miniCMV-SpCas9 (SpCas9) construct was generated by replacing the CMV promoter with a miniCMV promoter in the AAV-CMV-SpCas9 plasmid (Addgene #107024) via *Age*I and *Xba*I restriction enzyme sites. Other CRISPR/Cas endonucleases (SaCas9, Cas12a, and CjCas9) were subcloned from AAV-CMV:NLS-SaCas9-NLS-3xHA-bGHpA;U6: *Bsa*I-sgRNA (kindly provided by Feng Zhang; Addgene #61591), pcDNA3.1-hAsCpf1 (kindly provided by Feng Zhang; Addgene #69982) and CjCas9 (kindly provided by Feng Zhang; Addgene #68338) into AAV-CMV-SpCas9 plasmid by replacing SpCas9.

All-in-one single vector, AAV-miniCMV-SaCas9/YFP sgRNA or AAV-miniCMV-SaCas9/LacZ sgRNA were generated based on the AAV-CMV:NLS-SaCas9-NLS-3xHA-bGHpA;U6: *Bsa*I-sgRNA (kindly provided by Feng Zhang; Addgene #61591) by replacing the CMV promoter with miniCMV promoter, adding SpA terminator and inserting YFP sgRNA or LacZ sgRNA.

### Cell Culture and Transfection

HEK293A cells that stably express YFP (HEK293A-YFP) were generated as previously described (Hung et al., 2016, 2018). Cells were maintained in Dulbecco’s modified Eagle’s media (DMEM) (catalog no. 11965118; Life Technologies Australia, Mulgrave, VIC, Australia) supplemented with 10% fetal bovine serum (Sigma-Aldrich, St. Louis, MO, United States), 2 mM glutamine (catalog no. 2503008; Life Technologies Australia), antibiotic-antimycotic (catalog no. 15240062; Life Technologies Australia) in a humidified 5% CO_2_ atmosphere at 37°C. HEK293A-YFP cells were transfected with 750 ng of Cas endonuclease plasmid (under CMV promoter) and 750 ng of related sgRNA plasmid, or 750 ng of single SaCas9 plasmid, using lipofectamine 2000 (catalog no. 11668019; Life Technologies Australia), according to manufacturer’s instructions. YFP expression was evaluated 10 days later by collecting images of the cell cultures using a fluorescent microscope and by performing a flow cytometric analysis. Genomic DNA was extracted from cells after each treatment and used to carry out a T7 endonuclease 1 (T7E1) assay. The detailed information of reagents is provided in Supplementary Table S1.

For Cas endonuclease detection, HEK293A cells were transfected with 1000 ng of the Cas endonuclease plasmid (under the miniCMV promoter) or the CjCas9 plasmid (under CMV promoter), and protein lysates were generated 2 days later to perform a Western blot analysis.

### Genomic DNA Extraction and T7E1 Mismatch Detection Assay

Genomic DNA was extracted with QuickExtract DNA Extraction Solution (catalog no. QE09050; Lucigen, Biosearch technologies, Middleton, WI, United States) and used as the DNA template for PCR reactions performed using KAPA HiFi HotStart DNA Polymerase (catalog no. KR0369; Roche Diagnostics Australia, North Ryde, NSW, Australia) with primers listed in Supplementary Table S3 (CMV SeqFWD forward and EYFP SURVEYOR reverse primers). PCR products were then denatured at 95°C for 10 min and gradually lowered to room temperature to allow for DNA heteroduplex formation, which were then digested by T7 Endonuclease I (catalog no. M0302S; New England Biolabs, Ipswich, MA, United States) following the manufacturer’s instructions. The digested products were visualized on 2% (w/v) agarose gels.

### Western Blot Analysis

To validate that Cas protein expression was being driven effectively by the Cas endonuclease plasmids, HEK293A cells were transfected with AAV-miniCMV-SpCas9, AAV-miniCMV-SaCas9, AAV-miniCMV-Cas12a, AAV-miniCMV-CjCas9, and AAV-CMV-CjCas9 (under CMV promoter) plasmids. Cells were collected at day 2 post-transfection, and protein was extracted as described previously (Li et al., 2019). Protein samples were separated by using NuPAGE Electrophoresis system (Life Technologies Australia), after which proteins were transferred to polyvinylidene fluoride (PVDF) membranes (catalog no. 162-0177; Bio-Rad Laboratories; Hercules, CA, United States). Membranes were blocked with 5% (w/v) skim milk in TBS-T (10 mM Tris, 150 mM NaCl, and 0.05% Tween-20) at room temperature for 1 h and then incubated with a mouse monoclonal HA antibody (F-7) (1:500 dilution; catalog no. sc-7392; Santa Cruz Biotechnology, Dallas, TX, United States) or mouse monoclonal β-actin antibody (1:1000 dilution; catalog no. catalog no. MAB 1501; Merck Millipore, Burlington, MA, United States) at room temperature for 1 h. Membranes were washed, further incubated with a horseradish peroxidase-conjugated goat anti-mouse secondary antibody (1:5000 dilution; catalog no. A-11045; Life Technologies Australia) at room temperature for 1 h, and developed using the Amersham ECL Prime Western Blotting Detection Kit (catalog no. RPN2232; GE Healthcare Australia, Parramatta, NSW, Australia).

### Viral Production

The AAV7m8 vectors were prepared by transfecting HEK293D cells (kindly provided by Ian Alexander, Children’s Medical Research Institute, Australia) with the AAV-miniCMV-Cas (SpCas9, SaCas9, Cas12a, and CjCas9), AAV-CMV-CjCas9 or AAV-CMV-mCherry, selected YFP targeting sgRNAs or AAV-miniCMV-SaCas9/YFP sgRNA (all-in-one single vector) plasmids, helper plasmid (pXX6; kindly provided by Richard Samulski, The University of North Carolina School of Medicine, United States) and AAV7m8 capsid plasmid (Addgene #64839) using the calcium phosphate method (Hung et al., 2016). Viral vectors were purified using an AAVpro^®^ Purification Kit (All Serotypes) (catalog no. 6666; Clontech Laboratories, Mountain View, CA, United States) 48 h after viral transduction. Viral titrations were determined by real-time quantitative PCR using a Fast SYBR Green Master Mix (catalog no. 4385612; Life Technologies Australia) with AAV-ITR primers (Supplementary Table S3). The titrations of AAV7m8 were provided in Supplementary Table S4.

### Intravitreal Injection

Mice were anesthetized with an intraperitoneal injection of ketamine (60 mg/kg) and xylazine (10 mg/kg). Bioccular, intravitreal injections were performed under a surgical microscope, using a hand-pulled glass needle connected to a 10 μL Hamilton syringe (Bio-Strategy, Broadmeadows, VIC, Australia), as described previously (Hung et al., 2016, 2018). Eyes with severe surgical or post-operative complications such as ocular hemorrhage or inflammation were excluded from the study. A scleral incision was made on the nasal region with a 30G needle before the glass needle was inserted into the center of vitreous cavity to inject 1 μL of the dual vector system (2.5 × 10^9^vg AAV7m8-Cas endonuclease and 2.5 × 10^9^vg AAV7m8-YFP sgRNA), the SaCas9 single vector system (2.5 × 10^9^vg AAV7m8-miniCMV-SaCas9/YFP sgRNA and 2.5 × 10^9^vg AAV7m8-mCherry) or the control vector (2.5 × 10^9^vg AAV7m8-mCherry). A total of 150 YFP transgenic mice were randomly allocated to the following groups: mCherry control (*n* = 11), AAV7m8-miniCMV-SpCas9 (*n* = 12), AAV7m8-miniCMV-SaCas9 (*n* = 20), AAV7m8-miniCMV-Cas12a (*n* = 15), AAV7m8-CMV-CjCas9 (*n* = 11), AAV7m8-miniCMV-CjCas9 (*n* = 9) and AAV7m8-miniCMV-SaCas9/YFP sgRNA (*n* = 20), receiving the same viral injection regimen in each eye.

### Retinal Flat Mounts and Histology

Enucleated eyes were immersion fixed in ice-cold 4% (w/v) paraformaldehyde in PBS for 1 h before the retina was removed using a dissecting microscope as described previously (Hung et al., 2018). Processed retinal flat mounts were stained with NucBlue^TM^ Live ReadyProbes^TM^ Reagent (catalog no. R37605; Life Technologies Australia) for 20 min at room temperature before mounting with Dako Fluorescent mounting medium (catalog no. s3020; DAKO, Carpinteria, CA, United States). For histological assessment, eyes were fixed in 4% paraformaldehyde (w/v) in PBS for 1 h and embedded in optimal cutting temperature compound (Leica Biosystems, Germany) and stored at −80°C until cryosectioning. Serial 10 to 20-μm-thick cryosections were collected directly onto FLEX glass slides, followed by staining and mounting. Images of the retina were collected using an Olympus VS120 Slide Scanner or Perkin Elmer Spinning Disk Confocal Microscope (Zeiss spinning disk, Germany).

### Retinal Dissociation and Flow Cytometry Analysis

Retinas were rapidly dissected and digested using a papain dissociation kit (catalog no. LK003176; Worthington Biochemical Corporation, Lakewood, NJ, United States) following the manufacturer’s instructions to obtain a homogenous cell suspension. After dissociation, retinal cells were resuspended in FACS buffer (1% Bovine Serum Albumin in Phosphate Buffered Saline) and stained with DAPI (5 μg/mL; catalog no. D1306; Life Technologies Australia) to exclude dead cells. Dissociated retinal cells from C57BL/6 mice were used as a negative control for YFP expression. Live retinal cells with mCherry (532 nm, 622/22 nm) and/or YFP (488 nm, 513/26 nm) expression were detected by flow cytometry (MoFlo ASTRIOS; Beckman Coulter, Brea, CA, United States). We quantified the proportion of mCherry-labeled cells that co-labeled for YFP in each retina using FlowJo analysis software (FlowJo^®^; FlowJo LLC, Ashland, OR, United States). Eyes with severe surgical complications such as cataract or retinal detachment or those with negligible mCherry expression were excluded from the final FACS analysis.

### Statistical Analysis

GraphPad Prism7 software (GraphPad Software, Inc., La Jolla, CA, United States) was used for statistical analyses. The D’Agostino-Pearson test for normality was performed. Data are represented as mean ± SEM, and were analyzed using unpaired one-way analyses of variance (ANOVA). A value of *p* < 0.05 was considered statistically significant.

## Results

### *In vitro* YFP sgRNA Selection and Cas Endonuclease Validation

To select the most effective sgRNA for each Cas endonuclease, we first validated the on-target editing efficacy of different Cas endonucleases together with their respective sgRNAs using a T7E1 assay in HEK293A-YFP cells. Robust cleavage activity was evident in the groups transfected with the Cas endonuclease and their respective *YFP*-targeting sgRNAs, except for those treated with either CjCas9-*YFP* targeting constructs or *LacZ*-targeting controls (Figure 1B). Here, SpCas9-*YFP* targeting constructs were the most efficacious at knocking out *YFP* transgene expression, followed by Cas12a-*YFP* and SaCas9-*YFP* targeting constructs (Figure 1C).

The *YFP* disruption efficacy for each CRISPR/Cas construct was further quantified through flow cytometric analysis (Figure 1D). Compared to LacZ sgRNA counterparts, the percentage of *YFP*-expressing cells was significantly reduced by those transfected with SpCas9 and a *YFP*-targeting sgRNA (*YFP* sgRNA1: 26.0 ± 2.9%, *n* = 7, *p* < 0.0001; *YFP* sgRNA2: 11.5 ± 1.3%, *n* = 7, *p* < 0.0001; and *YFP* sgRNA3: 14.7 ± 2.9%, *n* = 7, *p* < 0.0001). Similarly, Cas12a-targeting conditions resulted in appreciable *YFP* transgene knockout with a preference for a 20 nt-protospacer (*YFP* sgRNA 20 nt: 33.6 ± 4.9%, *n* = 7, *p* < 0.0001; and 23 nt sgRNA: 55.0 ± 5.0%, *n* = 7, *p* < 0.0001). Comparatively, CjCas9 was less effective at abrogating *YFP* transgene expression (*YFP* sgRNA2: 69.5 ± 3.1%, *n* = 7, *p* = 0.0011) and failed to induce significant gene knockout in one of the conditions (CjCas9-*YFP* sgRNA1: 83.7 ± 0.7%, *n* = 7, *p* = 0.999); while there was no significant difference in editing efficiency (*p* = 0.9608) between the use of the SaCas9 single CRISPR construct (SaCas9/*YFP*-targeting sgRNA: 57.3 ± 3.2%, *n* = 4) and the dual CRISPR/Cas construct system (SaCas9 and its *YFP*-targeting sgRNA: 57.0 ± 2.0%, *n* = 7). The most effective *YFP*-targeting sgRNA for each Cas endonuclease (*YFP* sgRNA2 for SpCas9, 20 nt *YFP* sgRNA for Cas12a and *YFP* sgRNA2 for CjCas9) were selected for subsequent *in vivo* testing.

To validate the protein expression of HA-tagged Cas endonuclease in a recombinant AAV vector (driven by minimal promoter, miniCMV or full-length CMV promoter), HEK293A cells were transfected with AAV-miniCMV-SpCas9, AAV-miniCMV-SaCas9, AAV-miniCMV-Cas12a, AAV-miniCMV-CjCas9, and AAV-CMV-CjCas9. Cas endonuclease protein expression was evident with the use of the minimal promoter, except for AAV-miniCMV-CjCas9 (Supplementary Figure S1), which required the full-length CMV promoter to drive transgene expression. Therefore, four AAV-miniCMV-Cas endonucleases (SpCas9, SaCas9, and Cas12a) and the AAV-CMV-CjCas9 plasmid were used along with their selected sgRNAs for further *in vivo* CRISPR/Cas editing comparison.

### *In vivo* AAV7m8 Delivery of CRISPR/Cas in the Mouse Retina

**FIGURE 2:**
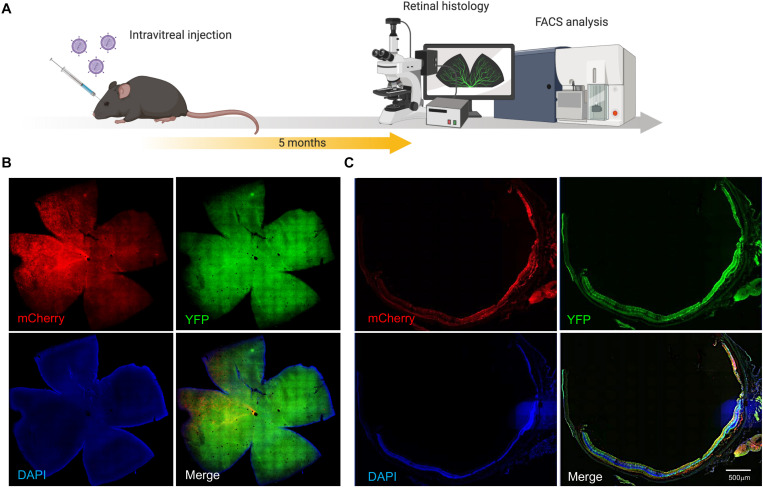
AAV7m8 mediated delivery of CRISPR/Cas to the mouse retina *in vivo.* **(A)** Schematic diagram of in vivo experiment. Mice were sacrificed 5 months after intravitreal injection. **(B)** Representative cross section image from retina co-transduced with AAV7m8-CRISPR/Cas and its selected YFP sgRNA. Mouse ID 29, right eye Robust AAV7m8 transductions in the retina were found. Scale bar: 200 μm. Images were taken by a Zeiss spinning disk confocal microscope. **(C)** Representative retinal whole-mount images from a mouse eye receiving AAV7m8-CRISPR/Cas and its selected YFP sgRNA. Mouse ID 76, right eye. Scale bar: 500 μm. Images were taken using an Olympus Slide Scanner. Please see Supplementary Figure S3 for representative cross-sectional images with higher magnification.

AAV7m8-mediated gene expression (mCherry) and distribution were assessed on retinal sectioning/flatmounts of the CMV-Cre:*Rosa26*-*YFP* mouse eye 5 months after intravitreal injection (Figure 2A). Retinal flatmount images from AAV7m8-CRISPR/Cas-injected retina showed robust expression of mCherry, although there was variation in fluorescence intensity across quadrants (Figure 2B). Fluorescence images revealed AAV7m8 transduction (as indicated by mCherry expression) was visible throughout the retina, including the ganglion cell layer (GCL), inner nuclear layer (INL) and even some parts of the retinal outer nuclear layer (ONL), with major expression within INL (Figure 2C and Supplementary Figure S3). Moreover, *YFP* expression could be found in all the layers of the retina with no observable difference between AAV7m8-CRISPR/Cas-treated mice and control mice.

### Comparison of YFP Knockout in the Mouse Retina With Different Cas Endonucleases Constructs

**FIGURE 3:**
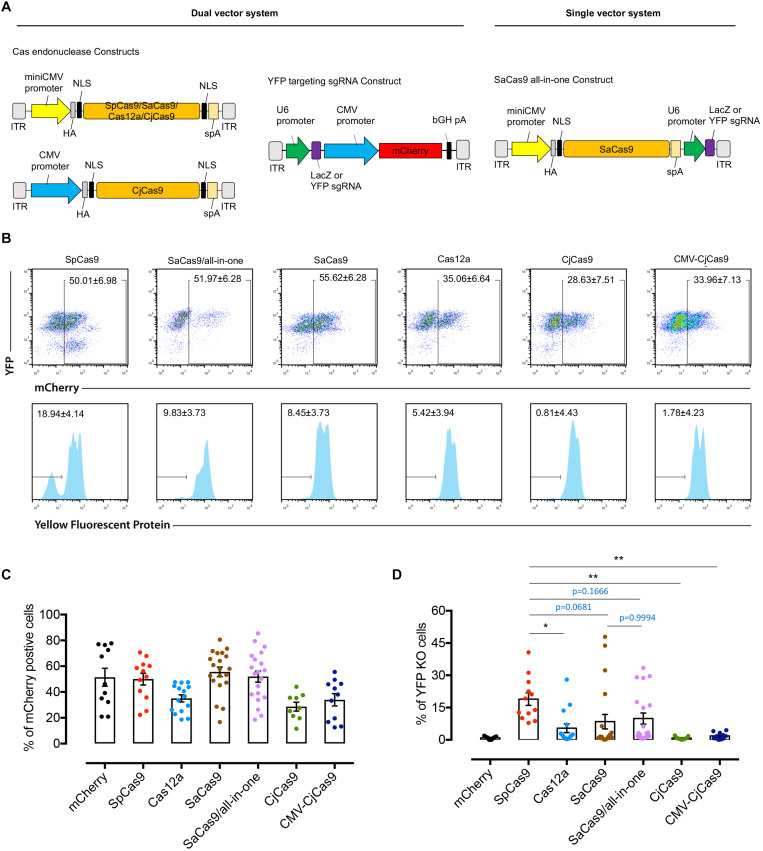
Comparison of YFP disruption in retinal cells with different CRISPR/Cas systems delivered by AAV7m8. **(A)** Schematic of the dual and single vector systems. For dual vector plasmids, the Cas endonuclease was driven by miniCMV or CMV promoter, whilst the sgRNA was driven by U6 promoter and mCherry under the control of CMV promoter to confirm vector transfection. For the single vector systems, an all-in-one plasmid with SaCas9 was designed with the Cas endonuclease being driven by a miniCMV promoter and sgRNA by U6 promoter. For Cas12a, we used the Cas endonuclease from *Acidaminococcus* (originally designated AsCpf1). A hemagglutinin (HA) tag was fused to the C-terminus of Cas endonuclease in the vector. **(B)** Representative FACS plots of dissociated retinal cells receiving different AAV7m8-CRISPR/Cas/AAV7m8-YFP sgRNA. The histograms in the lower panels (lower panel) were based on mCherry gating. Dissociated cells from one retina were used in each group. **(C)** Comparison of AAV7m8 transduction in the retina indicated by mCherry expression by FACS. Data are presented as mean ± SEM for 9–20 independent samples in each group. The D’Agostino-Pearson normality test was performed, and all groups were found to have a Gaussian distribution. Statistical analysis between groups was performed using one-way ANOVA followed by multiple comparisons test. **(D)** Comparison of *YFP* disruption in mCherry positive cells by FACS. Data are presented as mean ± SEM for 9–20 independent samples in each group. Data in two groups (Cas12a and Dual SaCas9) were found to not pass the D’Agostino-Pearson normality test, and as such the Kruskal–Wallis test was used. *p < 0.05, **p < 0.01, ***p < 0.001.

Five dual AAV7m8-CRISPR/Cas constructs (miniCMV-SpCas9, miniCMV-SaCas9, miniCMV-Cas12a, miniCMV-CjCas9, and CMV-CjCas9) with their selected *YFP*-targeting sgRNA and a single all-in-one AAV7m8-SaCas9 CRISPR construct (miniCMV-SaCas9/YFP-targeting sgRNA) were used to compare the editing efficacy in the retinal cell *in vivo* (Figure 3A). To evaluate and compare the *YFP* knockout *in vivo* delivered by AAV7m8-mediated different CRISPR/Cas system, the percentage of *YFP* disruption among mCherry positive retinal cells was quantified by flow cytometry (Figure 3B). The flow cytometric gating strategy is shown in Supplementary data (Supplementary Figure S2). Representative dot plots in Figure 3B illustrate the difference in *YFP* disruption in retinal cells receiving AAV7m8-SpCas9 CRISPR vector or control vector. Differences in AAV7m8 transduction efficiency were observed between CRISPR/Cas treatment groups (Figure 3C) with a lower percentage of mCherry positive cells observed in the retinas transfected with AAV7m8-Cas12a (35.1 ± 2.8%, *n* = 15) and AAV7m8-CjCas9 (28.6 ± 3.4%, *n* = 9) vectors. Retinas receiving AAV7m8-SpCas9 and AAV7m8-SaCas9 (both single and dual vector system) vectors had a relatively high proportion of mCherry expression (50.0 ± 4.6%, *n* = 12; 52.0 ± 4.3%, *n* = 20; 57.7 ± 3.3%, *n* = 19 respectively). For *YFP* disruption, AAV7m8-SpCas9 vector (18.9 ± 2.9%, *n* = 12) had the highest knockout efficiency of YFP among all the CRISPR/Cas systems, followed by AAV7m8-SaCas9 (single vector system: 8.4 ± 3.4%, *n* = 20; dual vector system: 9.8 ± 2.6%, *n* = 20) and Cas12a (5.4 ± 2.0%, *n* = 15), while AAV7m8-CjCas9 showed no disruption of *YFP* expression (Figure 3D). Moreover, there was no significant difference in the *YFP* disruption in the retinas receiving single and dual AAV7m8-SaCas9 vectors (single vector system: 8.4 ± 3.4% vs. dual vector system: 9.8 ± 2.6%, *n* = 20, *p* = 0.9994) (Figure 3D). Despite efficiency *in vivo* YFP knockout in animals administered AAV7m8-SpCas9, AAV7m8-SaCas9 and AAV7m8-Cas12a vectors, there was a high degree of variability between individual animals within identical treatment groups.

## Discussion

In this study, we provide a direct comparison of the efficacy for retinal editing *in vivo* with four different currently available CRISPR/Cas systems. Here, we showed that SpCas9 and Cas12a achieved better knockout efficiency than SaCas9 and CjCas9 *in vitro*. AAV7m8-packaged CRISPR/Cas construct with SpCas9 was found to have the highest editing efficacy among all Cas endonucleases *in vivo*. No significant difference in *YFP* gene editing was found between single and dual CRISPR/SaCas9 vector systems *in vitro* and *in vivo*.

This study was based on our previous work, which used AAV2-mediated delivery of CRISPR/Cas9 to achieve efficient gene editing in the inner layer of retina in Thy1-*YFP* mice (Hung et al., 2016). To assess and compare the genome efficiency in the whole retina, we applied a different murine model CMV-Cre:*Rosa26*-*YFP* transgenic mice (*YFP* mouse) which express YFP throughout the retina. To this end, we used the AAV7m8-pseudotype, an AAV2-based variant with enhanced retinal transduction when delivered through intravitreal injection (Dalkara et al., 2013; Khabou et al., 2016). As the degeneration of RPE and photoreceptors are involved in the majority of inherited retinal diseases, efficient gene delivery of CRISPR constructs to the outer layer of retina is imperative for therapeutic retinal gene editing. Subretinal injection of conventional AAVs (e.g., AAV2) has high photoreceptor transduction rate, but it is surgically challenging with more complications. In addition, the cellular transduction is confined within the injection bubble of the retina. mCherry expression was generally observed in at least two quadrants on retinal flat mount (Figure 2B), and any variation in distribution of transfection is likely to be due to stochastic or technical issues. Nonetheless, our study shows that AAV7m8-mediated CRISPR/Cas has reasonable pan-retinal transduction.

The stringent design of this study ensured a fair comparison of editing efficiency between different CRISPR/Cas systems. First, we analyzed the *YFP* coding sequence for all potential PAM sites for each Cas endonuclease and then designed sgRNA targeting *YFP* within a similar region. Previous work has shown that both guide RNA sequence and target gene-chromatin accessibility can directly influence CRISPR/Cas editing efficiencies. An important limitation of this work is the fact that we could not directly compare identical sequences across all endonucleases. Although we sought to target the same region within YFP, given their differing PAM requirements, each endonuclease had a different guide sequence, which may have directly biased editing efficiencies. Further, these PAM restrictions also limited the number of guide RNAs which could be directly tested. Naturally, there is a tradeoff between targeting the same genic region and ensuring similar *‘a priori’* guide RNA efficacy, and it is important to note that both factors should be considered in direct head-to-head comparisons. To further mitigate biases, we employed the same ubiquitous promoter (CMV for *in vitro* sgRNA selection, miniCMV for *in vivo*) for each endonuclease, and employed for the same virus. The only exception to this design was the use of the more potent CMV promoter for *in vivo* CjCas9 constructs, due to its poor expression on western blots of *in vitro* HEK293A cells. Despite this modification, CjCas9 barely demonstrated *YFP* knockout on flow cytometric analysis of *in vivo* specimens. We hypothesize that variation in CjCas9 codon-optimization may account for the differences observed in study compared to that reported by other groups (Kim et al., 2017).

We additionally found differences in gene knockout efficiency between *in vitro* and *in vivo* modes. For the *in vitro* study, SpCas9 outperformed Cas12a, followed by SaCas9 and CjCas9. For *in vivo* samples, SpCas9 remained the best-performing Cas endonuclease among all, without a clear trend among the other Cas orthologs. Initially, we hypothesized that the single all-in-one SaCas9 vector expressing both the SaCas endonuclease and its respective sgRNA may have a competitive or even higher editing efficiency compared to dual-vector mediated-editing with SpCas9, but we did not observe this result in our *in vivo* test.

In this proof-of-concept study, we engineered CMV-Cre:Rosa26-YFP mice. The principal advantage of screening a reporter gene at the Rosa26 locus, is that genomic edits could be readily quantified (using flow cytometry), at a single site. That is, only a single integration event would have occurred for our chosen reporter. Nevertheless, there are important limitations of this model, and it must be noted that Rosa26 locus is naturally permissive for DNA targeting. As such, our results may not be directly transferrable or representative for other endogenous genes, and likely represent the upper bounds for editing efficiencies.

In summary, we demonstrate that AAV7m8-mediated delivery of a SpCas9 construct appeard to achieve the most efficient gene modification in retinal cells *in vivo* among four currently available CRISPR/Cas systems. Ongoing research investigating different guide sequences at different loci is required before firm conclusions regarding retinal cell gene editing of different endonucleases can be made.

## Data Availability Statement

All datasets generated for this study are included in the article/Supplementary Material.

## Ethics Statement

The animal study was reviewed and approved by Animal Ethics Committees of the University of Tasmania (Reference Number A0014827).

## Author Contributions

FL, AH, G-SL, and SH conceptualized and designed the study. FL, KW, J-HW, CL, JB, JC, QW, QL, and MN performed all laboratory-based experiments. KY, RW, AP, and AC provided reagents and resources for this work. FL wrote the original draft, with all authors providing reviewing and editing. G-SL and AH jointly supervised this work. All authors contributed to the article and approved the submitted version.

## Conflict of Interest

The authors declare that the research was conducted in the absence of any commercial or financial relationships that could be construed as a potential conflict of interest.

## Footnotes

1 http://crispr.mit.edu

## Supplementary Material

FIGURE S1 The *in vitro* validation of Cas endonuclease expression.

FIGURE S2 Representative FACS plot showing gating strategy.

FIGURE S3 Representative retinal cross section with high magnification.

TABLE S1 Summary of reagents and resources used.

TABLE S2 Comparison of Cas orthologs for *in vivo* retinal gene editing application.

TABLE S3 Sequence of primers for sgRNA cloning, vector construction, sequencing and qPCR analysis.

TABLE S4 AAV7m8 titrations.

## References

[B1] Bakondi B. Lv W. Lu B. Jones M. K. Tsai Y. Kim K. J. (2016). In Vivo CRISPR/Cas9 gene editing corrects retinal dystrophy in the S334ter-3 rat model of autosomal dominant retinitis pigmentosa. *Mol. Ther.* 24 556–563. 10.1038/mt.2015.220 26666451 PMC4786918

[B2] Cong L. Ran F. A. Cox D. Lin S. Barretto R. Habib N. (2013). Multiplex genome engineering using CRISPR/Cas systems. *Science* 339 819–823. 23287718 10.1126/science.1231143 PMC3795411

[B3] Dalkara D. Byrne L. C. Klimczak R. R. Visel M. Yin L. Merigan W. H. (2013). In vivo-directed evolution of a new adeno-associated virus for therapeutic outer retinal gene delivery from the vitreous. *Sci. Transl. Med.* 5:189ra76. 10.1126/scitranslmed.3005708 23761039

[B4] Huang X. Zhou G. Wu W. Duan Y. Ma G. Song J. (2017). Genome editing abrogates angiogenesis in vivo. *Nat. Commun.* 8:112. 10.1038/s41467-017-00140-3 PMC5524639 28740073

[B5] Hung S. S. Li F. Wang J.-H. King A. E. Bui B. V. Liu G.-S. (2018). Methods for in vivo CRISPR/Cas editing of the adult murine retina. *Methods Mol. Biol.* 1715 113–133. 10.1007/978-1-4939-7522-8_9 29188510

[B6] Hung S. S. C. Chrysostomou V. Li F. Lim J. K. H. Wang J.-H. Powell J. E. (2016). AAV-mediated CRISPR/Cas gene editing of retinal cells in vivo. *Invest. Ophthalmol. Vis. Sci.* 57 3470–3476. 10.1167/iovs.16-19316 27367513

[B7] Ibraheim R. Song C.-Q. Mir A. Amrani N. Xue W. Sontheimer E. J. (2018). All-in-one adeno-associated virus delivery and genome editing by *Neisseria meningitidis* Cas9 in vivo. *Genome Biol.* 19:137. 10.1186/s13059-018-1515-0 PMC6146650 30231914

[B8] Jain A. Zode G. Kasetti R. B. Ran F. A. Yan W. Sharma T. P. (2017). CRISPR-Cas9-based treatment of myocilin-associated glaucoma. *Proc. Natl. Acad. Sci. U.S.A.* 114 11199–11204. 10.1073/pnas.1706193114 28973933 PMC5651749

[B9] Jarrett K. E. Lee C. De Giorgi M. Hurley A. Gillard B. K. Doerfler A. M. (2018). Somatic editing of Ldlr with adeno-associated viral-CRISPR is an efficient tool for atherosclerosis research. *Arterioscler. Thromb. Vasc. Biol.* 38 1997–2006. 10.1161/atvbaha.118.311221 30026278 PMC6202188

[B10] Jinek M. Chylinski K. Fonfara I. Hauer M. Doudna J. A. Charpentier E. (2012). A programmable dual-RNA-guided DNA endonuclease in adaptive bacterial immunity. *Science* 337 816–821. 10.1126/science.1225829 22745249 PMC6286148

[B11] Jo D. H. Koo T. Cho C. S. Kim J. H. Kim J.-S. Kim J. H. (2019). Long-Term effects of in vivo genome editing in the mouse retina using *Campylobacter jejuni* Cas9 expressed via adeno-associated virus. *Mol. Ther.* 27 130–136. 10.1016/j.ymthe.2018.10.009 30470629 PMC6318782

[B12] Khabou H. Desrosiers M. Winckler C. Fouquet S. Auregan G. Bemelmans A.-P. (2016). Insight into the mechanisms of enhanced retinal transduction by the engineered AAV2 capsid variant -7m8. *Biotechnol. Bioeng.* 113 2712–2724. 10.1002/bit.26031 27259396

[B13] Kim E. Koo T. Park S. W. Kim D. Kim K. Cho H.-Y. (2017). In vivo genome editing with a small Cas9 orthologue derived from *Campylobacter jejuni*. *Nat. Commun.* 8:14500. 10.1038/ncomms14500 PMC5473640 28220790

[B14] Koo T. Park S. W. Jo D. H. Kim D. Kim J. H. Cho H.-Y. (2018). CRISPR-LbCpf1 prevents choroidal neovascularization in a mouse model of age-related macular degeneration. *Nat. Commun.* 9:1855. 10.1038/s41467-018-04175-y PMC5945874 29748595

[B15] Latella M. C. Di Salvo M. T. Cocchiarella F. Benati D. Grisendi G. Comitato A. (2016). In vivo editing of the human mutant rhodopsin gene by electroporation of plasmid-based CRISPR/Cas9 in the Mouse Retina. *Mol. Ther. Nucleic Acids* 5:e389. 10.1038/mtna.2016.92 27874856 PMC5155324

[B16] Li F. Hung S. S. C. Mohd Khalid M. K. N. Wang J.-H. Chrysostomou V. Wong V. H. Y. (2019). Utility of self-destructing CRISPR/Cas constructs for targeted gene editing in the retina. *Hum. Gene Ther.* 30 1349–1360. 10.1089/hum.2019.021 31373227

[B17] Maeder M. L. Stefanidakis M. Wilson C. J. Baral R. Barrera L. A. Bounoutas G. S. (2019). Development of a gene-editing approach to restore vision loss in Leber congenital amaurosis type 10. *Nat. Med.* 25 229–233. 30664785 10.1038/s41591-018-0327-9

[B18] Mali P. Yang L. Esvelt K. M. Aach J. Guell M. DiCarlo J. E. (2013). RNA-guided human genome engineering via Cas9. *Science* 339 823–826. 10.1126/science.1232033 23287722 PMC3712628

[B19] Pan X. Philippen L. Lahiri S. K. Lee C. Park S. H. Word T. A. (2018). In Vivo Ryr2 editing corrects *Catecholaminergic* polymorphic ventricular tachycardia. *Circ. Res.* 123 953–963. 30355031 10.1161/CIRCRESAHA.118.313369 PMC6206886

[B20] Ran F. A. Cong L. Yan W. X. Scott D. A. Gootenberg J. S. Kriz A. J. (2015). In vivo genome editing using *Staphylococcus aureus* Cas9. *Nature* 520 186–191. 10.1038/nature14299 25830891 PMC4393360

[B21] Ruan G.-X. Barry E. Yu D. Lukason M. Cheng S. H. Scaria A. (2017). CRISPR/Cas9-mediated genome editing as a therapeutic approach for leber congenital amaurosis 10. *Mol. Ther.* 25 331–341. 10.1016/j.ymthe.2016.12.006 28109959 PMC5368591

[B22] Swiech L. Heidenreich M. Banerjee A. Habib N. Li Y. Trombetta J. (2015). In vivo interrogation of gene function in the mammalian brain using CRISPR-Cas9. *Nat. Biotechnol.* 33 102–106. 10.1038/nbt.3055 25326897 PMC4492112

[B23] Tsai Y.-T. Wu W.-H. Lee T.-T. Wu W.-P. Xu C. L. Park K. S. (2018). Clustered regularly interspaced short palindromic repeats-based genome surgery for the treatment of autosomal dominant retinitis pigmentosa. *Ophthalmology* 125 1421–1430. 10.1016/j.ophtha.2018.04.001 29759820 PMC6109419

[B24] Xia C.-H. Ferguson I. Li M. Kim A. Onishi A. Li L. (2018). Essential function of NHE8 in mouse retina demonstrated by AAV-mediated CRISPR/Cas9 knockdown. *Exp. Eye Res.* 176 29–39. 10.1016/j.exer.2018.06.026 29958869 PMC9236278

[B25] Xu L. Lau Y. S. Gao Y. Li H. Han R. (2019). Life-long AAV-mediated CRISPR genome editing in dystrophic heart improves cardiomyopathy without causing serious lesions in mdx Mice. *Mol. Ther.* 27 1407–1414. 10.1016/j.ymthe.2019.05.001 31129119 PMC6697345

[B26] Yu W. Mookherjee S. Chaitankar V. Hiriyanna S. Kim J.-W. Brooks M. (2017). Nrl knockdown by AAV-delivered CRISPR/Cas9 prevents retinal degeneration in mice. *Nat. Commun.* 8:14716. 10.1038/ncomms14716 PMC5355895 28291770

