# Supplementary material for: Comparison of CRISPR/Cas Endonucleases for in vivo Retinal Gene Editing
Source: Front Cell Neurosci. 2020 Sep 10;14:570917. doi: 10.3389/fncel.2020.570917 (PMC7511709; doi:10.3389/fncel.2020.570917)

**SUPPLEMENTARY MATERIALS**

**Table S1.** Comparison of Cas orthologs for *in vivo* retinal gene editing application.

**Table S2.** Sequence of primers for sgRNA cloning, vector construction, sequencing and qPCR analysis.

**Table S3.** AAV7m8 titrations.

**Figure S1**. The *in vitro* validation of Cas endonuclease expression.

**Figure S2.** Representative FACS plot showing gating strategy.

**Figure S3.** Representative retinal cross section with high magnification.

**Table S1. Summary of reagents and resources used.**

| **REAGENT or RESOURCE** | **SOURCE** | **IDENTIFIER** |
| --- | --- | --- |
| **Antibodies** | | |
| HA-probe Antibody (F-7) | Santa Cruz Biotechnology | sc-7392 |
| mouse monoclonal β-actin antibody | Millipore | MAB 1501 |
| HRP-conjugated goat anti-mouse secondary antibody | Life Technologies Australia | A-11045 |
| **Chemicals** | | |
| Sodium azide (NaN3) | Sigma-Aldrich | S2002-100g |
| Trizma base | Sigma-Aldrich | T1503-1KG |
| Glycine | Sigma-Aldrich | G8898-1KG |
| NaH_2_PO_4_ | Sigma-Aldrich | S0751-500G |
| Calcium chloride dihydrate (CaCl_2_• 2H_2_O) | Sigma-Aldrich | 223506-500G |
| Agarose | Sigma-Aldrich | A9539-250G |
| LB broth base | Life Technologies Australia | 12780-029 |
| LB broth with agar | Sigma-Aldrich | L2897-1KG |
| DAPI | Life Technologies Australia | D1306 |
| **Critical Commercial Assays** | | |
| TaKaRa AAVpro Purification Kit (All Serotypes) | Clontech Laboratories | 6666 |
| Fast SYBR Green Master Mix | Life Technologies Australia | 4385612 |
| Amersham ECL Prime Western Blotting Detection Kit | GE Healthcare Australia | RPN2232 |
| QIAquick PCR purification kit (250) | Qiagen | 28106 |
| Qiaprep spin miniprep kit (250) | Qiagen | 27106 |
| HiSpeed Plasmid Maxi Kit (10) | Qiagen | 12262 |
| QIAfilter Plasmid Mega Kit (5) | Qiagen | 12281 |
| **Experimental Models: Cell Lines** | | |
| Human embryonic kidney (HEK) 293A | Life Technologies Australia | R70507 |
| HEK293A-YFP | Guei-Sheung Liu Lab | n/a |
| HEK293D | Ian Alexander Lab | n/a |
| **Experimental Models: Organisms/Strains** | | |
| CMV-Cre::Rosa26-YFP transgenic mice | University of Tasmania | n/a |
| **Plasmids** | | |
| AAV-U6-sgRNA-hSyn-mCherry | Feng Zhang Lab | Addgene #87916 |
| pX601-AAV-CMV::NLS-SaCas9-NLS-3xHA-bGHpA;U6::BsaI-sgRNA | Feng Zhang Lab | Addgene #61591 |
| pY010 (pcDNA3.1-hAsCpf1) | Feng Zhang Lab | Addgene #69982 |
| pX404-CjCas9 | Feng Zhang Lab | Addgene #68338 |
| AAV-CMV-SpCas9 | Alex Hewitt Lab | Addgene #107024 |
| pXX6 | Richard Samulski Lab | n/a |
| 7m8 | John Flannery & David Schaffer Lab | Addgene #64839 |
| **Softwares and Algorithms** | | |
| ImageJ version 1.48 | Schneider et al., 2012 | https://imagej.nih.gov/ij |
| GraphPad Prism7 | GraphPad Software | http://graphpad.com |
| Adobe Photoshop (CC 2017.1.1) | Adobe | https://adobe.com |
| Guide RNA design | Feng Zhang Lab | http://crispr.mit.edu |
| Guide RNA design | Benchling | https://benchling.com |
| FlowJo | FlowJo LLC | https://flowjo.com |
| **Others** | | |
| Dulbecco's modified Eagle's media (DMEM) | Life Technologies Australia | 11965118 |
| Iscove's Modified Dulbecco's Medium (IMDM) | Life Technologies Australia | 12440061 |
| Glutamine | Life Technologies Australia | 2503008 |
| Antibiotic-antimycotic | Life Technologies Australia | 15240062 |
| Opti-MEM | Life Technologies Australia | 11058021 |
| Lipofectamine 2000 transfection reagent | Life Technologies Australia | 11668019 |
| Virkon (5gm tablet) | MedShop Australia | MED1610661 |
| AgeI | New England Biolabs | R0552L |
| XbaI | New England Biolabs | R0145L |
| T7 Endonuclease I (T7E1) | New England Biolabs | M0302S |
| QuickExtract DNA Extraction Solution | Lucigen | QE09050 |
| KAPA HiFi HotStart DNA Polymerase | Roche Diagnostics Australia | KR0369 |
| Polyvinylidene fluoride (PVDF) membranes | Bio-Rad Laboratories | 162-0177 |
| TE buffer (PH 8.0, 500 mL) | Life Technologies Australia | AM9849 |
| Sodium Dodecyl Sulfate (SDS) | Life Technologies Australia | 15525017 |
| Tween 20 | Sigma-Aldrich | P9416-50ML |
| SYBR Safe-DNA Gel Stain | Life Technologies Australia | S33102 |
| 1KB plus DNA ladder | Life Technologies Australia | 10787018 |
| Gel loading Dye Purple (6x) | New England Biolabs | B7025S |
| NuPAGE® LDS Sample Buffer (4X) | Life Technologies Australia | 0007 |
| Novex® sharp pre-stained protein standard | Life Technologies Australia | LC5800 |
| Cell Lysis Buffer | Thermo Fisher Scientific | 89900 |
| T4 DNA ligase | New England Biolabs | M0202S |

# Table S2. Comparison of main Cas orthologs for *in vivo* retinal gene editing applications

| **Cas orthologs** | **origins** | **PAM**  **(5’-3’)** | **Size (Kb)** | ***In vivo* retinal gene editing applications Reference** |
| --- | --- | --- | --- | --- |
| SpCas9 | *Streptococcus pyogenes* | NGG | *~4.2* | [(Bakondi et al., 2016; Hung et al., 2016; Latella et al., 2016; Huang et al., 2017; Jain et al., 2017; Ruan et al., 2017; Yu et al., 2017; Tsai et al., 2018; Li et al., 2019)](https://paperpile.com/c/EDDS7e/qXkFd+WeeQ+bbV6T+iJ93E+10xmy+zwgTR+S9hX5+XjVmf+Yspyb) |
| SaCas9 | *Staphylococcus aureus* | NNGRRT | *~3.2* | [(Maeder et al., 2019)](https://paperpile.com/c/EDDS7e/oI7BM) |
| CjCas9 | *Campylobacter jejuni* | NNNNRYAC,  NNNNACA | *~2.9* | [(Kim et al., 2017; Jo et al., 2019)](https://paperpile.com/c/EDDS7e/RVNYK+qX8e9) |
| Cas12a  (Cpf1) | *Acidaminococcus*, *Lachnospiraceae* | TTTN | ~3.9 (AsCpf1)  ~3.7 (LbCpf1) | [(Koo et al., 2018)](https://paperpile.com/c/EDDS7e/gAZ70) |
| NmCas9 | *Neisseria meningitidis* | NNNNGATT | *~3.2* | [(Xia et al., 2018)](https://paperpile.com/c/EDDS7e/Jpig5) |

# Table S3. Sequence of primers for sgRNA cloning, vector construction, sequencing and qPCR analysis

| **Primer name** | **Sequence** | **Purpose** |
| --- | --- | --- |
| YFP sgRNA4 top (SpCas9) | ACCGCCGTCCAGCTCGACCAGGAT | cloning |
| YFP sgRNA4 btm (SpCas9) | AACATCCTGGTCGAGCTGGACGGC | cloning |
| YFP sgRNA5 top (SpCas9) | ACCGCCGTCCAGCTCGACCAGGA | cloning |
| YFP sgRNA5 btm (SpCas9) | AACATCCTGGTCGAGCTGGACGGC | cloning |
| YFP sgRNA6 top (SpCas9) | ACCGCGTCGCCGTCCAGCTCGACC | cloning |
| YFP sgRNA6 btm (SpCas9) | AACGGTCGAGCTGGACGGCGACGC | cloning |
| LacZ sgRNA1 top (SpCas9) | ACCTGCGAATACGCCCACGCGAT | cloning |
| LacZ sgRNA1 btm (SpCas9) | AACATCGCGTGGGCGTATTCGCA | cloning |
| YPF sgRNA 20nt top (Cas12a) | ACCGTAATTTCTACTCTTGTAGATCGTCGCCGTCCAGCTCGACCAGGTTTTGGCC | cloning |
| YPF sgRNA 20nt btm (Cas12a) | AAAAGGTCGAGCTGGACGGCGACGATCTACAAGAGTAGAAATTAC | cloning |
| YPF sgRNA 23nt top (Cas12a) | ACCGTAATTTCTACTCTTGTAGATCGTCGCCGTCCAGCTCGACCTTTTGGCC | cloning |
| YPF sgRNA 23nt btm (Cas12a) | AAAAGGTCGAGCTGGACGGCGACGATCTACAAGAGTAGAAATTACC | cloning |
| LacZ sgRNA 20nt top (Cas12a) | ACCGTAATTTCTACTCTTGTAGATCGAATACGCCCACGCGATGGTTTTGGCC | cloning |
| LacZ sgRNA 20nt btm (Cas12a) | AAAACCATCGCGTGGGCGTATTCGATCTACAAGAGTAGAAATTAC | cloning |
| LacZ sgRNA 23nt top (Cas12a) | ACCGTAATTTCTACTCTTGTAGATCGAATACGCCCACGCGATGGGTATTTTGGCC | cloning |
| LacZ sgRNA 23nt btm (Cas12a) | AAAATACCCATCGCGTGGGCGTATTCGATCTACAAGAGTAGAAATTACC | cloning |
| YFP sgRNA (SapI) top (SaCas9) | ACCGTACGTCGCCGTCCAGCTCGAC | cloning |
| YFP sgRNA (SapI) btm (SaCas9) | AACGTCGAGCTGGACGGCGACGTAC | cloning |
| LacZ sgRNA (SapI) top (SaCas9) | ACCGCTTTGCGAATACGCCCACGCG | cloning |
| LacZ sgRNA (SapI) btm (SaCas9) | AACCGCGTGGGCGTATTCGCAAAGC | cloning |
| YFP sgRNA1 top (CjCas9) | ACCGCGAGCTGGACGGCGACGTAAAC | cloning |
| YFP sgRNA1 btm (CjCas9) | AACGTTTACGTCGCCGTCCAGCTCGC | cloning |
| YFP sgRNA2 top (CjCas9) | ACCGTCGCCGTCCAGCTCGACCAGG | cloning |
| YFP sgRNA2 btm (CjCas9) | AACCCTGGTCGAGCTGGACGGCGAC | cloning |
| LacZ sgRNA top (CjCas9) | ACCGTTGCGAATACGCCCACGCGATG | cloning |
| LacZ sgRNA btm (CjCas9) | AACCATCGCGTGGGCGTATTCGCAAC | cloning |
| SpA F | aattcAATAAAAGATCTTTATTTTCATTAGATCTGTGTGTTGGTTTTTTGTGTA | cloning |
| SpA R | CGCGTACACAAAAAACCAACACACAGATCTAATGAAAATAAAGATCTTTTATTG | cloning |
| (gB1) U6_Cas12a_sgRNA_scaffold | GCTAGCGGTACCACGCGTGAGGGCCTATTTCCCATGATTCCTTCATATTTGCATATACGATACAAGGCTGTTAGAGAGATAATTGGAATTAATTTGACTGTAAACACAAAGATATTAGTACAAAATACGTGACGTAGAAAGTAATAATTTCTTGGGTAGTTTGCAGTTTTAAAATTATGTTTTAAAATGGACTATCATATGCTTACCGTAACTTGAAAGTATTTCGATTTCTTGGCTTTATATATCTTGTGGAAAGGACGAAACACCGTAATTTCTACTCTTGTAGATGGAAGAGCGAGCTCTTCTTTTTGGGCCCGCGGCCGCGAATTC | cloning |
| (gB2) U6_SaCas9_sgRNA_scaffold | GCTAGCGGTACCACGCGTGAGGGCCTATTTCCCATGATTCCTTCATATTTGCATATACGATACAAGGCTGTTAGAGAGATAATTGGAATTAATTTGACTGTAAACACAAAGATATTAGTACAAAATACGTGACGTAGAAAGTAATAATTTCTTGGGTAGTTTGCAGTTTTAAAATTATGTTTTAAAATGGACTATCATATGCTTACCGTAACTTGAAAGTATTTCGATTTCTTGGCTTTATATATCTTGTGGAAAGGACGAAACACCGGAAGAGCGAGCTCTTCTGTTTTAGTACTCTGGAAACAGAATCTACTAAAACAAGGCAAAATGCCGTGTTTATCTCGTCAACTTGTTGGCGAGATTTTTGGGCCCGCGGCCGCGAATTC | cloning |
| (gB3) U6_CjCas9_sgRNA_scaffold | GCTAGCGGTACCACGCGTGAGGGCCTATTTCCCATGATTCCTTCATATTTGCATATACGATACAAGGCTGTTAGAGAGATAATTGGAATTAATTTGACTGTAAACACAAAGATATTAGTACAAAATACGTGACGTAGAAAGTAATAATTTCTTGGGTAGTTTGCAGTTTTAAAATTATGTTTTAAAATGGACTATCATATGCTTACCGTAACTTGAAAGTATTTCGATTTCTTGGCTTTATATATCTTGTGGAAAGGACGAAACACCGGAAGAGCGAGCTCTTCTGTTTTAGTCCCTGAAGGGACTAAAATAAAGAGTTTGCGGGACTCTGCGGGGTTACAATCCCCTAAAACCGCTTTTTTGGGCCCGCGGCCGCGAATTC | cloning |
| Kozak Cas12a AgeI F | CGCACCGGTgccaccATGACACAGTTCGAGGGCTT | cloning |
| AsCpf1 EcoRI R | CGCGAATTCTTAGGCATAGTCGGGGACAT | cloning |
| CMV-ApaI F | GCGGGGCCCCGTTACATAACTTACGGTAAATGGC | cloning |
| CMV-KpnI R | GCGGGTACCTCTGACGGTTCACTAAACGAGC | cloning |
| miniCMV_CjCas9 FWD | ACGATGTTCCAGATTACGCTTCGCCGAAGAAAAAGCGCAA | cloning |
| miniCMV_CjCas9 REV | AAAGATCTTTTATTGAATTCTTAGCTGGCCTCCACCTTTC | cloning |
| miniCMV-XbaI/AgeI F | CTAGATAATACGACTCACTATAGGGGGATCCACGTATGTCGAGGTAGGCGTGTACGGTGGGAGGCCTATATAAGCAGAGCTCGTTTAGTGAACCGTCAGATCGCCTGGAGGTACCGCCACCA | cloning |
| miniCMV-XbaI/AgeI R | CCGGTGGTGGCGGTACCTCCAGGCGATCTGACGGTTCACTAAACGAGCTCTGCTTATATAGGCCTCCCACCGTACACGCCTACCTCGACATACGTGGATCCCCCTATAGTGAGTCGTATTAT | cloning |
| AAV-ITR FW | GGAACCCCTAGTGATGGAGTT | qPCR |
| AAV-ITR REV | CGGCCTCAGTGAGCGA | qPCR |
| CMV Seq‐FWD | CGCAAATGGGCGGTAGGCGTG | T7E1 PCR |
| EYFP SURVEYOR REV | CTGGTAGCTCAGGTAGTGGTTG | T7E1 PCR |
| U6 primer (for sequencing the gRNA) | GAGGGCCTATTTCCCATGATTCC | sequencing |
| miniCMV seq primer | GTACGGTGGGAGGCCTATATAA | sequencing |

#

#

# Table S4. AAV7m8 titrations

| **AAV7m8** | **Vector** | **Titration (vg/mL)** |
| --- | --- | --- |
| AAV7m8-SpCas9 | AAV-miniCMV-SpCas9 | 5.13x10^14^ |
| AAV7m8-SaCas9 | AAV-miniCMV-SaCas9 | 5.48x10^14^ |
| AAV7m8-Cas12a | AAV-miniCMV-Cas12a | 6.22x10^14^ |
| AAV7m8-CjCas9 (CMV) | AAV-CMV-CjCas9 | 1.61x10^15^ |
| AAV7m8-CjCas9 (miniCMV) | AAV-miniCMV-CjCas9 | 3.53x10^14^ |
| AAV7m8-YFP sgRNA2 (SpCas9) | AAV-CMV-mCherry-YFP sgRNA2  (SpCas9) | 2.31x10^14^ |
| AAV7m8-YFP sgRNA 20nt (Cas12a) | AAV-CMV-mCherry-YFP sgRNA 20nt  (Cas12a) | 1.06x10^15^ |
| AAV7m8-YFP sgRNA (SaCas9 dual) | AAV-CMV-mCherry-YFP sgRNA  (SaCas9) | 1.22x10^14^ |
| AAV7m8-YFP sgRNA2 (CjCas9) | AAV-CMV-mCherry-YFP sgRNA2  (CjCas9) | 1.26x10^15^ |
| AAV7m8-YFP sgRNA (SaCas9 single) | AAV-miniCMV-SaCas9-U6-YFP sgRNA | 2.55x10^14^ |
| AAV7m8-mCherry | AAV-CMV-mCherry | 1.58x10^14^ |

#

**Figure S1. Cas endonuclease validation.** *In vitro* validation of different Cas endonucleases by western blot. Representative western blot of Cas protein expression in HEK293A cells treated with AAV-Cas plasmids two days after transfection. CjCas9 expression was not detectable in cells transfected with AAV-miniCMV-CjCas9 plasmid by western blot with HA tag antibody. β-actin was used as a loading control.

**
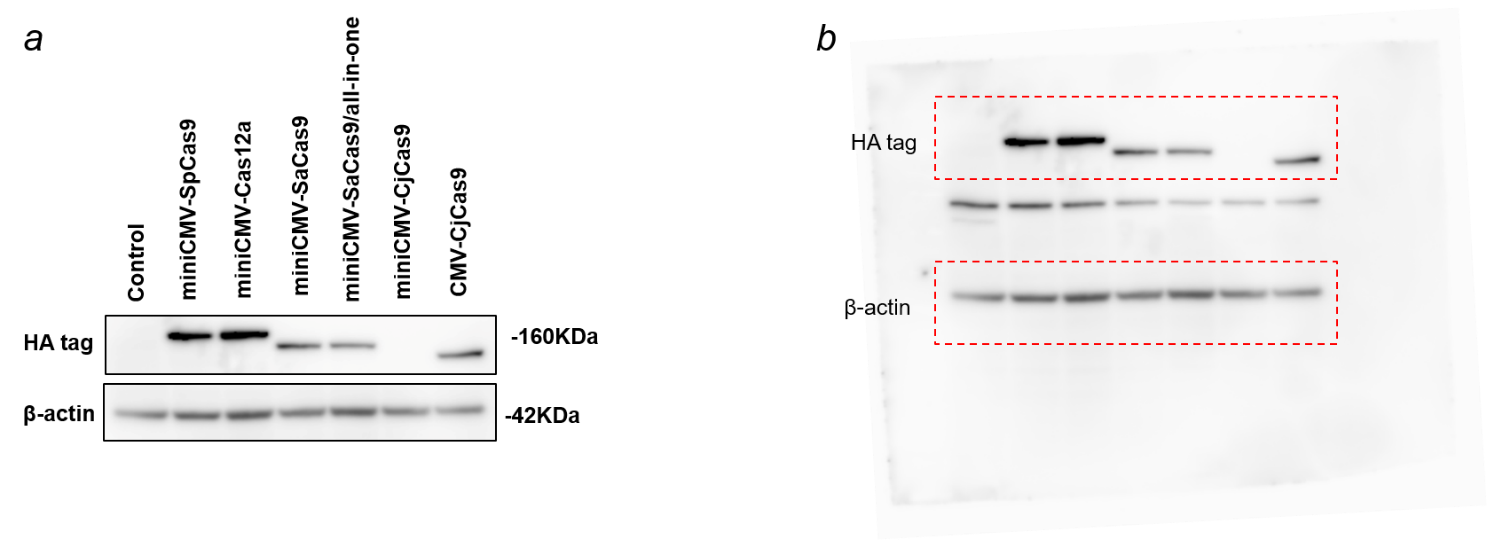
**

**Figure S2. Representative FACS plot showing gating strategy.** Single cells from dissociated retina were gated on forward scatter (FSC-H)/side scatter (SSC-A) plot and live cells were further gated on DAPI. Retinal cells were freshly dissociated and quantified using a flow cytometer. Forward versus side scatter (FSC vs SSC) gating was used to exclude cellular debris. DAPI staining was further applied to exclude dead cells. For each sample, 10,000 cells were counted automatically.


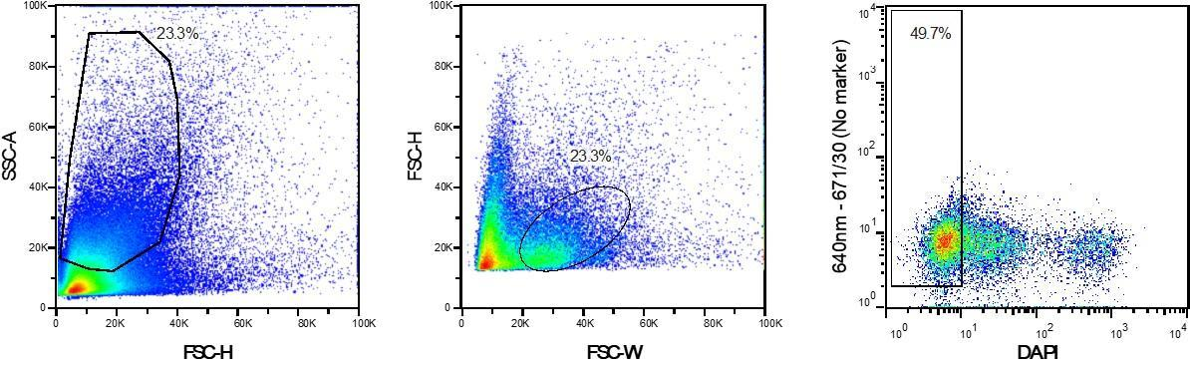


**Figure S3. Representative retinal cross-sectional image with high magnification.** AAV7m8-mediated delivery of mCherry to the inner and outer retina following intravitreal injection. Abbreviations: GCL, ganglion cell layer; INL, inner nuclear layer; ONL, outer nuclear layer.


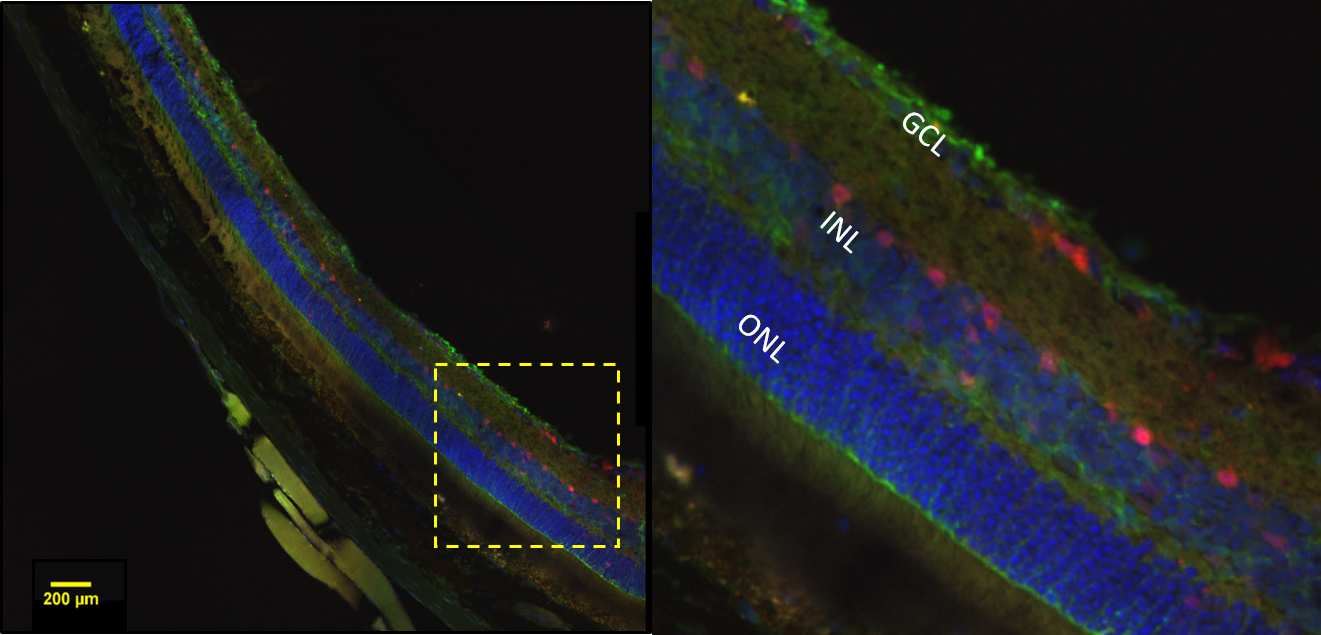

Supplement: FIGURE S1 — The in vitro validation of Cas endonuclease expression. [file Data_Sheet_1.docx]
